# Production of the Ramoplanin Activity Analogue by Double Gene Inactivation

**DOI:** 10.1371/journal.pone.0154121

**Published:** 2016-05-05

**Authors:** Jungang Han, Junsheng Chen, Lei Shao, Junliang Zhang, Xiaojing Dong, Pengyu Liu, Daijie Chen

**Affiliations:** 1 State Key Laboratory of New Drug and Pharmaceutical Process, Shanghai Institute of Pharmaceutical Industry, 1320 West Beijing Rd., Shanghai 200040, China; 2 School of Pharmacy, Shanghai Jiao Tong University, 800 Dongchuan Rd., Minhang District, Shanghai 200240, China; Rockefeller University, UNITED STATES

## Abstract

Glycopeptides such as vancomycin and telavancin are essential for treating infections caused by Gram-positive bacteria. But the dwindling availability of new antibiotics and the emergence of resistant bacteria are making effective antibiotic treatment increasingly difficult. Ramoplanin, an inhibitor of bacterial cell wall biosynthesis, is a highly effective antibiotic against a wide range of Gram-positive bacteria, including methicillin-resistant *Staphylococcus aureus*, vancomycin-intermediate resistant *Clostridium difficile* and vancomycin-resistant *Enterococcus sp*. Here, two tailoring enzyme genes in the biosynthesis of ramoplanin were deleted by double in-frame gene knockouts to produce new ramoplanin derivatives. The deschlororamoplanin A2 aglycone was purified and its structure was identified with LC-MS/MS. Deschlororamoplanin A2 aglycone and ramoplanin aglycone showed similar activity to ramoplanin A2. The results showed that α-1,2-dimannosyl disaccharide at Hpg^11^ and chlorination at Chp^17^ in the ramoplanin structure are not essential for its antimicrobial activity. This work provides new precursor compounds for the semisynthetic modification of ramoplanin.

## Introduction

Antibiotic resistance has increasingly become more serious in the clinic. The β-lactamase production in Gram-positive bacteria render several β-lactam antibiotics ineffective. In the United States more than 94,000 people are infected and about 19,000 are killed by methicillin-resistant *Staphylococcus aureus* (MRSA) every year. As “The last line of resistant Gram-positive bacterial antibiotic”, vancomycin also was seriously challenged with the emergence of vancomycin-resistant *Enterococci* (VRE) in recent years [1]. Ramoplanin (1) which was isolated from the fermentation broth of *Actinoplanes sp*. ATCC33076 more than 20 years ago is a highly effective antibiotic against a wide range of Gram-positive bacteria, including MRSA, vancomycin-intermediate resistant *Clostridium difficile* and VRE [2,3,4]. Ramoplanin inhibits of the biosynthesis of bacterial cell wall. Previous studies have shown that ramoplanin interferes with the transglycosylase-catalyzed extracellular polymerization of Lipid II to block the transglycosylation step of peptidoglycan biosynthesis [5,6]. As an oral antibiotic, ramoplanin is currently is in phase III clinic trials for the treatment of *Clostridium difficile* infection (CDI) and prevention of VRE infection [7].

**Fig 1:**
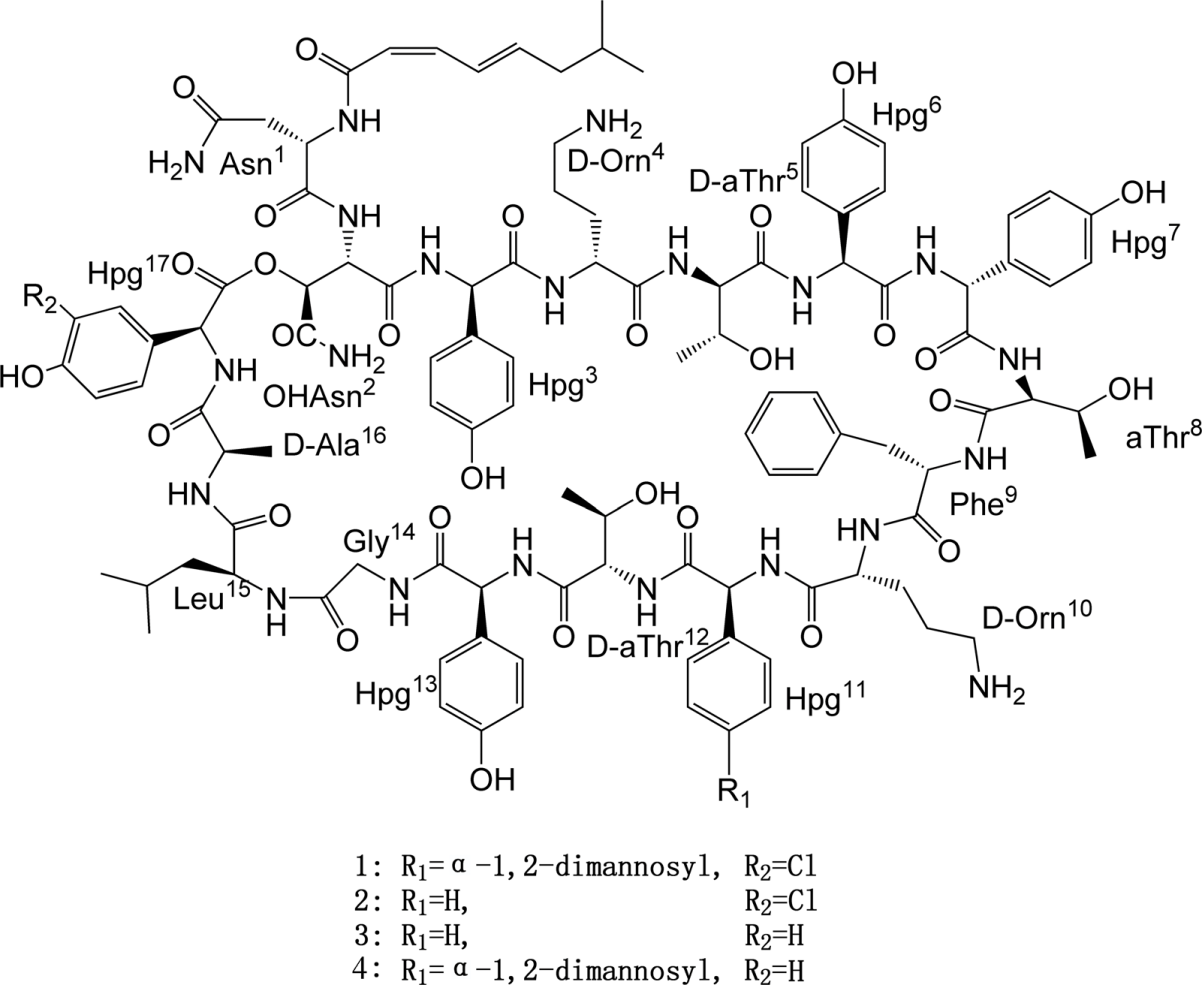
Structure of ramoplanin and its derivative.

Structurally, ramoplanin, a lipoglycodepsipeptide compound, contains 17 amino acids joined by a lactone linkage between a beta hydroxyl group on a lactone acid 2 (OHAsn^2^) and the carboxyl terminus of amino acid 17 (Chp^17^) (Fig 1). Ramoplanin contains an α-1,2-dimannosyl group attached by an α-glycosidic linkage to the phenol of amino acid 11 and a monochlorinated hydroxyphenylglycine in position 17. This residue is one of the three hydrophobic groups containing the Chp^17^-Hpg^3^-Phe^9^ aryl core. Non-ribosomal peptide synthetases (NRPSs) catalyzed the biosynthesis of Non-ribosomal peptides (NRPs) by the consecutive condensation of amino acids and other carboxylic acids in bacteria [8,9]. The ramoplanin biosynthesis gene cluster was isolated and analyzed from *Actinoplanes sp*. ATCC33076 in 2002 [10]. The analysis showed that the consecutive action of four NRPSs (*ram12*, *13*, *14* and *17*) assembled the peptide core of ramoplanin containing a mixture of L and D amino acids (nine L, seven D) as well as several nonproteinogenic side chains [11]. To investigate the glycosylation mechanism in ramoplanin biosynthesis *ram29* was inactivated and mutant strain *A*.*CJS1001* was shown to produce the ramoplanin aglycone (2), suggesting that Ram29 is responsible for incorporation of the mannose sugars in ramoplanin [12]. Herein we described the double inactivation of *ram29* gene coding mannosyltransferase and *ram20* gene coding halogenase in *Actinoplanes sp*. ATCC33076 and identification of ramoplanin derivative.

## Materials and Methods

### Materials, DNA isolation, manipulations and sequencing

Bacterial strains and plasmids used in this study are listed in Table 1. *Actinoplanes sp*. ATCC33076, *E*.*coli DH5*α, *E*. *coli* ET12567, pKC1139 plasmid and Deschlororamoplanin A2 (4) were obtained from standard commercial sources. DNA isolation and manipulation were performed by standard methods [13,14]. Primer synthesis and DNA sequencing were performed at Shanghai Mapbio Biotechnology Co.

**Table 1:** Strains and plasmids used in this work.

Strains or plasmids	Relevant features	Source or reference
Strains		
*Actinoplanes*		
ATCC33076	Wild type; ramoplanin producer	This Laboratory
*A*. *CJS1001*	ATCC33076 derivative, *with the ram29* gene in-frame deleted	[12]
*A*.*CSL1015*	*A*. *CJS1001* derivative *with the ram20* gene in-frame deleted	This work
*E*. *coli*		
DH5α	General cloning host	Invitrogen
ET12567	Methylation deficient; Donor strain for conjugation between *E*.*coli* and *Actinoplanes*	[15]
Plasmids		
pSP72	*E*. *coli* general cloning vector, Amp^R^	Promega
pKC1139	*E*. *coli—Streptomyces* shuttle vector; *rep*^*ts*^; *OriT*, Am^R^	[16]
pJG1011	1.1-kb PCR fragment containing the upstream of *ram20* in pSP72	This work
pJG1012	1.0-kb PCR fragment containing the downstream of *ram20* in pSP72	This work
pJG1013	1.1-kb left arm and 1.0-kb right arm of *ram20* in pKC1139	This work

### Inactivation of *ram20* in *A*.*CJS1001* and genotype analysis of mutant

To inactivate *ram20* in A. CJS1001, two homolog arms containing the upstream and downstream of *ram20* were PCR amplified from *Actinoplanes sp*. ATCC33076 genomic DNA and inserted into the shuttle vector pKC1139. The 1.1-kb fragment was amplified using primers 5’-AAAAAGCTTCGACGATGACGATCCGTGTCC-3’ and 5’-AAATCTAGACGAGGTGGCGAAGGTGAA-3’ and cloned into the *Hin*dIII and *Xba*I sites of pSP72 to generate pJG1011. The 1.1-kb fragment was amplified with primers 5’-AAATCTAGACAGGAGAAGGCGCTGTTCGAG-3’ and 5’-AAAGAATTCCGTTCCTGCTGACCAACAAGGT-3’ using ATCC33076 genomic DNA as a template. The purified PCR product was then digested with *Eco*RI and *Xba*I and then inserted into appropriately digested pSP72 to yield pJG1012. The identity of the PCR product with two homolog exchange arms (GenBank accession number AX417445) was confirmed by sequencing. The 1.1-kb *Hin*dIII/*Xba*I fragment and 1.1-kb *Eco*RI/*Xba*I fragment were then excised from pJG1011 and pJG1012 respectively, and inserted into pKC1139 prepared by digestion with *Hin*dIII and *Eco*RI to construct the in-frame inactivation plasmid pJG1013.

The resultant pJG1013 was then introduced into *A*. *CJS1001* from *E*.*coli* ET12567 by intergeneric conjugation as previously described. For inactivation of *ram20*, apramycin-resistant colonies at 37°C were identified as the positive integrated mutants, with a single-crossover homologous recombination. These mutants were further cultured for three days in 3 ml liquid tryptic soy broth (TSB) for five rounds without apramycin. The genotypes of resultant apramycin-sensitive strains were confirmed by PCR amplification and sequencing, which lead to identification of the recombinant strain *A*.*CSL1015*, in which the 534bp internal fragment was in-frame deleted.

### Production and preparation of ramoplanin and its derivative in fermentation cultures

*Actinoplanes sp*. ATCC 33076 and its mutant strains were fermented as previously described [12,17]. The fermentation broth was adjusted to pH 3.0 and extracted twice with an equal volume of ethyl alcohol. The crude extract was applied to WQ NO.1 macroporous resin. The 30% ethanol eluent containing the target compound was concentrated using an Agilent reversed-phase column (ZORBAX Eclipse Plus C18 9.4×250mm, 5μm). The mobile phase was 0.4% ammonium formate/acetonitrile (68:32 v/v). The peaks containing the target compound were collected. The high density ammonium formate was eliminated using hydrophilic C18 packing. The 70% methanol eluent containing ramoplanin derivative was collected and dried. The highly purified ramoplanin derivative was used for analysis and bioassay.

### Analysis of ramoplanin and its derivative

The fractions of ramoplanin and its derivative were detected by HPLC during the separation and purification processes performed on an Agilent reversed-phase column (ZORBAX Eclipse Plus C18 4.6×250mm, 5μm) using 0.1% ammonium formate/acetonitrile (65:35 v/v) as the mobile phases at 0.8 ml/min and 231nm. LC-MS and Tandem MS analyses were performed on LTQ XL combined with Agilent 1260 HPLC by the Instrumental Analysis Center of Shanghai Jiaotong University. The LC-MS^2^ analyses with this instrument were performed with a Water CORTECS C18 2.1mm*100mm, 2.7μm column, using H_2_O/CH_3_CN (80:20) as mobile phaseand with the ionization source run in positive mode. An MS^2^ analysis was performed by selecting the doubly-charged ions (1116 for ramoplanin A2 aglycone and 1099 for deschlororamoplanin A2 aglycone respectively) as parent ions.

### Evaluation of antibacterial activity

The activies of ramoplanin and its analogs against MRSA and VRE were determined using a liquid microdilution method. MIC values were detected according to the CLSI guidelines [18]. ATCC 25923 (*methicillin-sensitive Staphylococcus aureus*, MSSA), ATCC 43300 (*methicillin-resistant Staphylococcus aureus*, MRSA), ATCC 29212 (*Enterococcus faecalis*) and ATCC 51299 (*vancomycin-resistant Enterococcus faecalis*, VRE) were employed in this study. Ramoplanin and its analogs were dissolved in 30% methyl alcohol, which was also used as the negative control during the serial tests.

## Results

### Generation of the *ram29* and *ram20* double in-frame inactivated mutant

**Fig 2:**
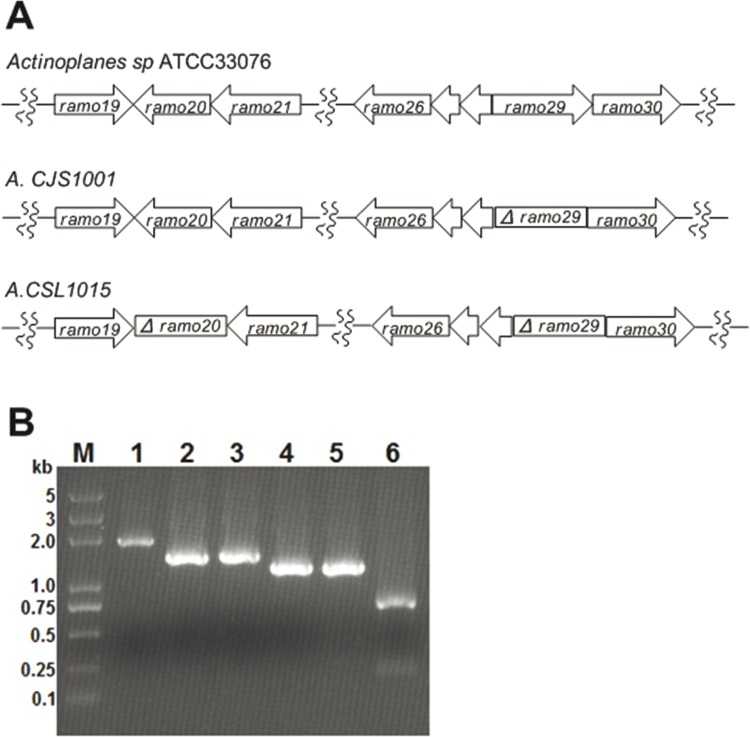
Genotypes of recombinant strains and PCR analysis with the genomic DNA from *Actinoplanes sp*. ATCC33076 and its mutation as the template. (A) Genotypes of ATCC33076 and its mutants; (B) Using 5’- ATGGATGTCCCGAGGGTG -3’ and 5’- TCAGGGCTTGTCGCATCG -3’ as primers, ram29 in-frame deletion PCR analysis of recombinant strains confirmed that they have the designed genotypes that the double-crossover homologous recombination event took place: 1.8 kb (*lane 1*) for wild type, 1.4 kb (*lane 2*) for *A*.*CJS1001*, and 1.4 kb (*lane 3*) for *A*.*CSL1015*. Using 5’-ATCTCGCTGAGCACGGTGAAGG-3’ and 5’- TGCACGCCGGAGGAGAACAC -3’ as primers, *ram20* in-frame deletion PCR analysis of recombinant strains confirmed that they have the designed genotypes that the double-crossover homologous recombination event took place: 1.2 kb (*lane 4*) for wild type, 1.2 kb (*lane 5*) for *A*.*CJS1001*, and 0.75kb (*lane 6*) for *A*.*CSL1015*.

Two homolog exchange arms for inactivation of *ram20*, containing the upstream and downstream of *ram20*, were PCR amplified from *Actinoplanes sp*. ATCC33076 genomic DNA. The identity of the PCR product was confirmed by sequencing. A *ram20* knockout strain of *A*. *CJS1001* was constructed by double-crossover in-frame deletion of *ram20* gene using pJG1013 to create strain *A*.*CSL1015*. The *ram20* gene was inactivated by introducing a 456-bp in-frame deletion at the center of the gene to avoid any effects on the transcription of upstream and downstream *ram20*. PCR analysis of the genomic DNAs from the recombinant strains *A*. *CJS1001* and *A*.*CSL1015* showed that the *ram29* and *ram20* double in-frame inactivated mutant was successfully constructed (Fig 2).

### Production and preparation of ramoplanin and its derivative in fermentation cultures

**Fig 3:**
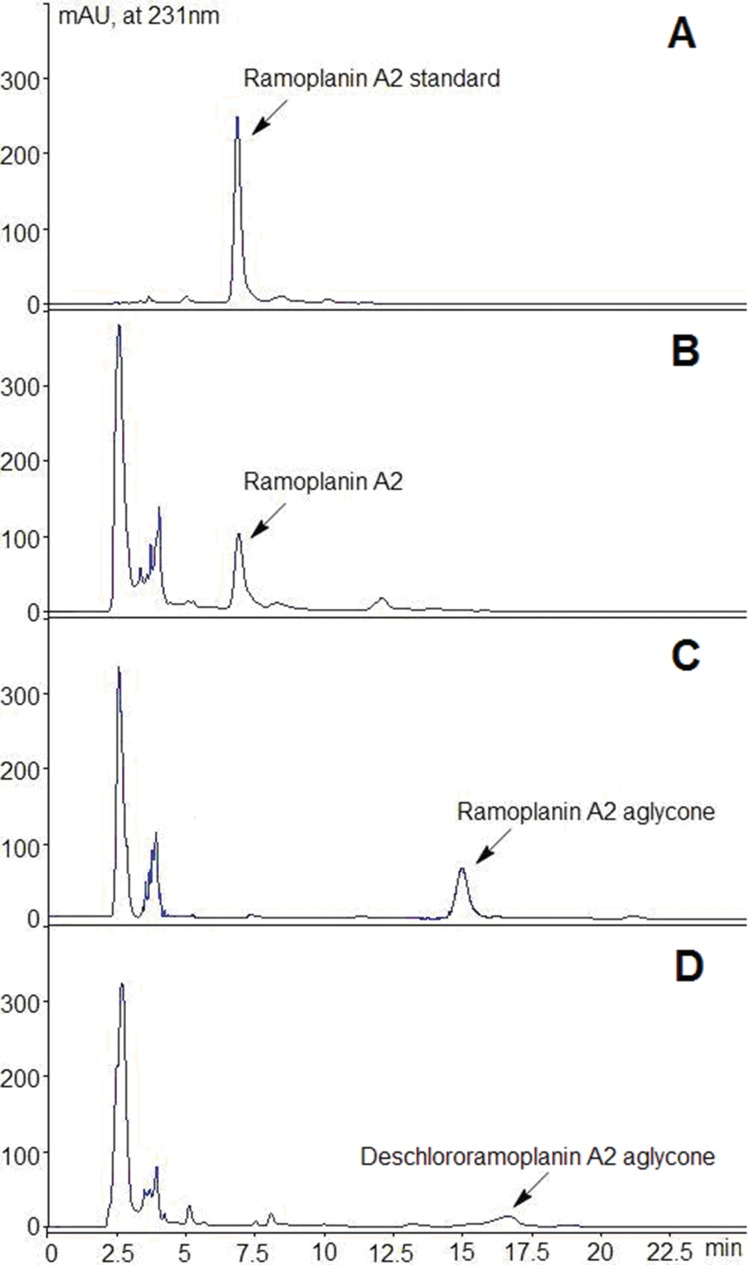
HPLC analysis of ramoplanin and its derivative. (A) Ramoplanin A2 standard; (B) Production of ramoplanin A2 in *Actinoplanes sp*. ATCC33076; (C) Ramoplanin derivative production in *A*.*CJS1001*; (D) Ramoplanin derivative production in *A*.*CSL1015*.

*Actinoplanes sp*. ATCC33076, *A*. *CJS1001* and *A*.*CSL1015* were grown in ramoplanin production medium under the same conditions for producing ramoplanin. Efficient culture cleanup for ramoplanin A2 and its derivative was performed on the fermentation broth of *Actinoplanes sp*. ATCC33076 and recombinant strains. HPLC of the sample revealed that the peaks of ramoplanin A2 and ramoplanin A2 aglycone were eliminated and a new analog, named ramoplanin X, with longer retention time than ramoplanin A2 and ramoplanin A2 aglycone for *A*.*CSL1015* (Fig 3). According to the standard curve of ramoplanin A2, its concentration in the broth of *Actinoplanes sp*. ATCC33076 was about 500 μg/ml. However, the yield of ramoplanin A2 aglycone was 100 μg/ml in the broth of *A*. *CJS1001*. Following isolation and purification from the *A*.*CSL1015* culture broth, 5 mg of purified ramoplanin X was obtained.

### Identification of ramoplanin derivative

**Fig 4:**
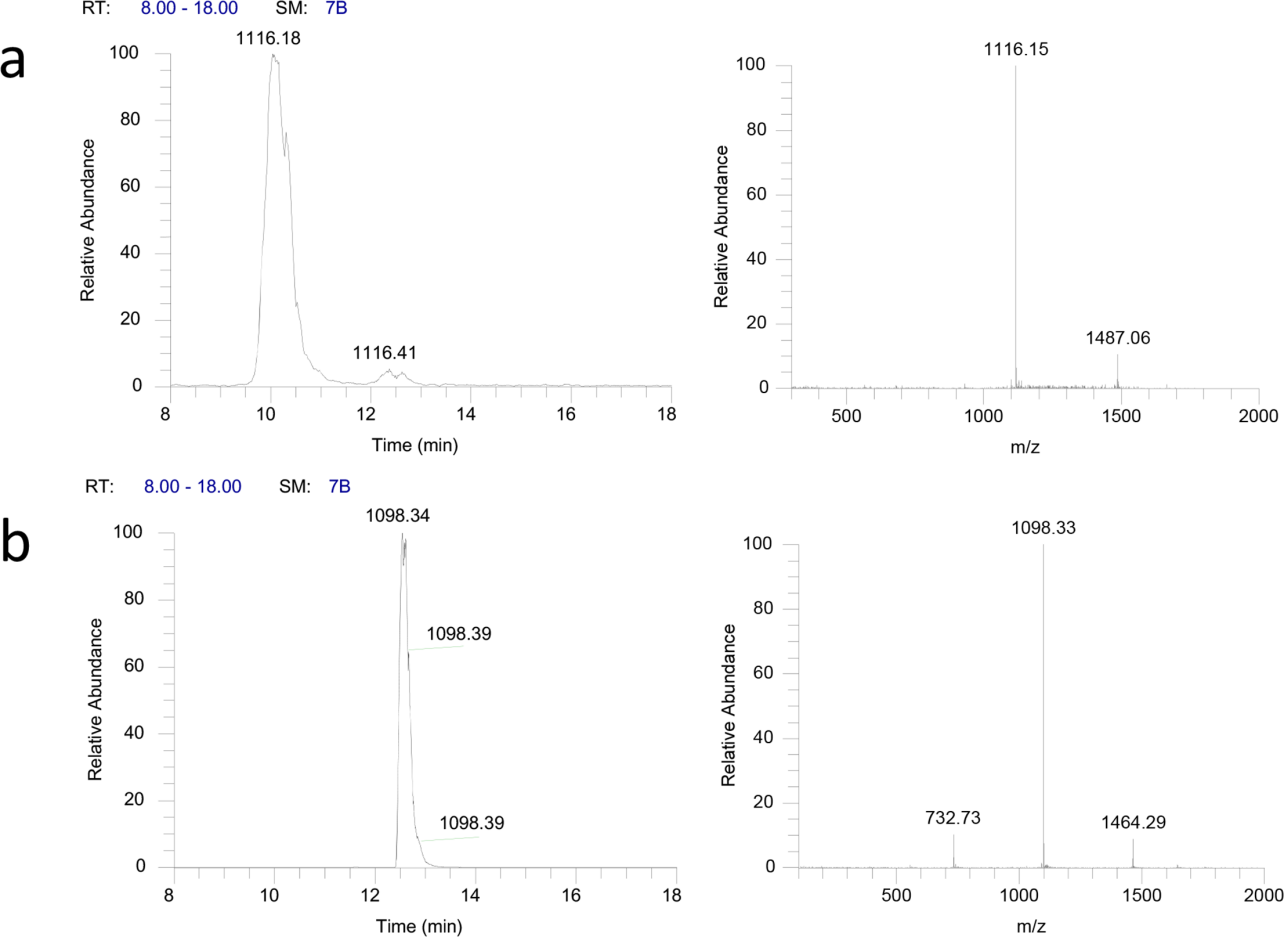
LC-MS analysis of the metabolites produced by mutants. (A) The doubly charged ions for ramoplanin A2 aglycone (m/z 1116) produced by *A*.*CJS1001*; (B) The doubly charged ions for deschlororamoplanin A2 aglycone (m/z 1098) produced by *A*.*CSL1015*. The left panel shows the base peak corresponding to the compounds while the right panel indicates the spectrum.

As compared to *A*.*CJS1001*, *A*.*CSL1015* did not produce no ramoplanin A2 aglycone m/z ([M+2H]^2+^) = 1116 but a new major product, ramoplanin X, m/z ([M+2H]^2+^) = 1099, which was 17Da less than the ion obtained with ramoplanin A2 aglycone (Fig 4).

**Fig 5:**
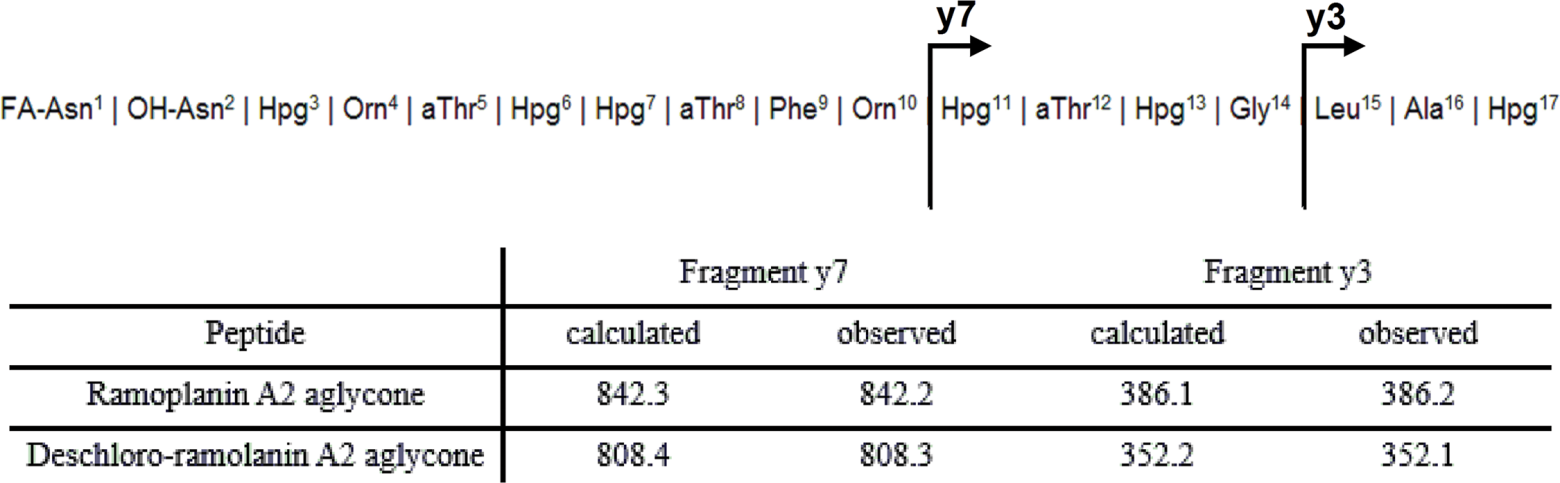
Localization of the missing chlorine atom of deschlororamoplanin A2 aglycone produced by *A*.*CSL1015*.

The loss of 17Da may be attributed to the loss of the chlorine atom to the Hpg^17^. To confirm this, the purified product as well as ramoplanin A2 aglycone was subjected to a tandem MS analysis. In the MS^2^ spectra of ramoplanin A2 aglycone, fragment peptides y7 (Hpg^11^-Cl-Hpg^17^, m/z 842.2) and y3 (Leu^15^-Cl-Hpg^17^, m/z 386.2) were observed, while in the MS^2^ spectra of the purified peptide produced by *A*.*CSL1015*, the fragment ions for y7 and y3 were 34Da less than the corresponding ions in ramoplanin A2 aglycone (Fig 5). This clearly indicates the Hpg^17^ in the peptide produced by *A*.*CSL1015* was not halogenated. The peptide produced by *A*.*CSL1015* was confirmed as deschlororamoplanin A2 aglycone (3).

### Antibacterial activity of ramoplanin derivative

Antibacterial activities of purified deschlororamoplanin A2 aglycone were compared with ramoplanin A2 and ramoplanin A2 aglycone. The MICs of ramoplanin A2, ramoplanin A2 aglycone and deschlororamoplanin A2 aglycone against ATCC 25923 (*methicillin-sensitive Staphylococcus aureus*, MSSA), ATCC 43300 (*methicillin-resistant Staphylococcus aureus*, MRSA), ATCC 29212 (*Enterococcus faecalis*) and ATCC 51299 (*vancomycin-resistant Enterococcus faecalis*, VRE) were determined. The results revealed that deschlororamoplanin A2 aglycone had equipotent activity of ramoplanin A2 against MRSA and VRE (Table 2).

**Table 2:** Antibacterial activity of ramoplanin and purified its derivative.

Microorganism	MIC (μg/ml)				
	Ramoplanin A2	Ramoplanin A2 aglycone	Deschlororamoplanin A2 aglycone	Deschlororamoplanin A2	Enduracidin
*Staphylococcus aureus* ATCC43300	4	2	4	2	8
*Staphylococcus aureus* ATCC25923	2	2	2	2	8
*Enterococcus faecalis* ATCC29212	1	0.5	1	1	8
*Enterococcus faecalis* ATCC51299	1	1	1	1	8

## Discussion

Nonribosomal peptides (NRPs) synthesized by non-ribosomal peptide synthetases are a diverse family of natural products with a broad range of biological activities and pharmacological properties. NRPs are often potent antibiotics, antifungals, antitumor, immunosuppressants, antiviral or antiparasitic therapeutics for clinical use [19]. The structural modification of NRPs is an important component of the natural product drug development process. Because of ramoplanin’s favorable antibacterial properties, efforts have been made to generate ramoplanin analogs by total or semi-synthesis of the natural compound [20,21,22]. But the complexity of ramoplanin structure makes chemical approaches difficult. Combinatorial biosynthesis can be defined as the application of genetic engineering to modify biosynthetic pathways of natural products in order to produce new and altered structures [23].

Previous studies showed that ramoplanin inhibited the intracellular glycosyltransferase (MurG)-catalyzed conversion of Lipid I (undecaprenyl-pyrophospho-N-acetylmuramyl-pentapeptide) to Lipid II (undecaprenyl-pyrophospho-Nacetylmuramyl-N-acetylglucoseamine- pentapeptide) and blocked the transglycosylation step of peptidoglycan biosynthesis by interfering with the transglycosylase-catalyzed extracellular polymerization of Lipid II [5,24]. Ramoplanin contains an N-acyl chain which may insert into the bacterial membrane phospholipid bilayers and two positively charged ornithines (Orn) at positions 4 and 10 which may interact with anionic phospholipids that predominate in Gram-positive bacteria. Later studies showed the significance of disrupting the Chp^17^-Asn^2^ lactone bond on antimicrobial activity, the requirement for cationic charge on ramoplanin’s two orns, and the importance of presentation of the Hpg^3^-Orn^10^ PG binding sequence in a conformationally restrained manner [25,26,27,28].

Glycosylation is a frequently occurring NPRs antibiotics modification that was proven to be related to antibiotics activity. Chlorination is hypothesized to improve NPR dimerization that may in turn positively enhance antimicrobial activity [29,30]. Halogen in antibiotics also plays a role in determining adverse effects. For example, a halogen at position 8 in sparfloxacin and clinafloxacin caused serious phototoxicity [31]. In our studies, the deletion of α-1,2-dimannosyl disaccharide attached to Hpg^11^ and chlorination at Chp^17^ of ramoplanin had no effect on its bioactivity against methicillin-resistant *Staphylococcus aureus* (MRSA) and vancomycin-resistant *Enterococcus sp*. (VRE) suggesting that α-1,2-dimannosyl disaccharide at Hpg^11^ and chlorination at Chp^17^ of ramoplanin are not essential for its antimicrobial activity. The deletion of disaccharide group will increase lipid solubility and the logarithm of the octanol/water partition coefficient (LogP) of ramoplanin in favor of the permeability of the cell membrane. These will benefit the absorption and distribution of drugs in the body. The deletion of chlorine at Chp17 reduces the risk of potential toxicity. So, the fermentation products of mutants *A*. *CJS1001* and *A*.*CSL1015*, ramoplanin aglycone and deschlororamoplanin A2 aglycone, also provide new precursor compounds for the semi-synthetic modification of ramoplanin. Halogenation and glycosylation of biosynthetic products may serve as powerful methods to introduce novel functionality into small molecules and may be used in combinatorial biosynthetic approaches in drug discovery.
